# Shotgun sequencing of *Yersinia enterocolitica *strain W22703 (biotype 2, serotype O:9): genomic evidence for oscillation between invertebrates and mammals

**DOI:** 10.1186/1471-2164-12-168

**Published:** 2011-03-31

**Authors:** Thilo M Fuchs, Katharina Brandt, Mandy Starke, Thomas Rattei

**Affiliations:** 1 Lehrstuhl für Mikrobielle Ökologie, Department Biowissenschaften, Wissenschaftszentrum Weihenstephan, Technische Universität München, Weihenstephaner Berg 3, 85354 Freising, Germany; 2 University of Vienna, Department of Computational Systems Biology, Althanstrasse 14, 1090 Vienna, Austria

## Abstract

**Background:**

*Yersinia enterocolitica *strains responsible for mild gastroenteritis in humans are very diverse with respect to their metabolic and virulence properties. Strain W22703 (biotype 2, serotype O:9) was recently identified to possess nematocidal and insecticidal activity. To better understand the relationship between pathogenicity towards insects and humans, we compared the W22703 genome with that of the highly pathogenic strain 8081 (biotype1B; serotype O:8), the only *Y. enterocolitica *strain sequenced so far.

**Results:**

We used whole-genome shotgun data to assemble, annotate and analyse the sequence of strain W22703. Numerous factors assumed to contribute to enteric survival and pathogenesis, among them osmoregulated periplasmic glucan, hydrogenases, cobalamin-dependent pathways, iron uptake systems and the *Yersinia *genome island 1 (YGI-1) involved in tight adherence were identified to be common to the 8081 and W22703 genomes. However, sets of ~550 genes revealed to be specific for each of them in comparison to the other strain. The plasticity zone (PZ) of 142 kb in the W22703 genome carries an ancient flagellar cluster Flg-2 of ~40 kb, but it lacks the pathogenicity island YAPI_Ye_, the secretion system *ysa *and *yts1*, and other virulence determinants of the 8081 PZ. Its composition underlines the prominent variability of this genome region and demonstrates its contribution to the higher pathogenicity of biotype 1B strains with respect to W22703. A novel type three secretion system of mosaic structure was found in the genome of W22703 that is absent in the sequenced strains of the human pathogenic *Yersinia *species, but conserved in the genomes of the apathogenic species. We identified several regions of differences in W22703 that mainly code for transporters, regulators, metabolic pathways, and defence factors.

**Conclusion:**

The W22703 sequence analysis revealed a genome composition distinct from other pathogenic *Yersinia enterocolitica *strains, thus contributing novel data to the *Y. enterocolitica *pan-genome. This study also sheds further light on the strategies of this pathogen to cope with its environments.

## Background

The genus *Yersinia *currently comprises three human pathogens (*Y. pestis*, *Y. pseudotuberculosis*, and *Y. enterocolitica*), and at least 14 species considered harmless for humans, namely *Y. aldovae*, *Y. bercovieri*, *Y. frederiksenii*, *Y. intermedia*, *Y. kristensenii*, *Y. mollaretii*, *Y. rohdei*, *Y. ruckeri *[1], *Y. aleksiciae *[2], *Y. similis *[3], *Y. massiliensis *[4], *Y. entomophaga *[5], *Y. nurmii *[6] and *Y. pekkanenii *[7]. *Y. enterocolitica *infection causes diarrhea, terminal ileitis, and mesenteric lymphadenitis, but not systemic infection, and often leads to secondary immunologically induced sequelae including erythema nodosum, reactive arthritis and Reiter's syndrome [8]. The heterogenous species *Y. enterocolitica *encompass six biotypes as differentiated upon biochemical tests [9]. Biotype 1A strains are considered avirulent due to the lack of the *Yersinia *virulence plasmid pYV [10], whereas biotype 1B strains are highly pathogenic and lethal for mice. They form a geographically distinct group predominately isolated in North America and carry a high-pathogenicity island (HPI) [11]. Biotype 2-5 strains mainly found in Europe and Japan compose a weakly pathogenic group unable to kill mice [12].

We have recently shown that strain W22703 (biotype 2, serotype O:9) confers lethality towards nematodes and *Manduca sexta *larvae upon oral infection, and that this insecticidal activity is correlated with the presence of the so-called pathogenicity island TC-PAI_Ye _[13,14]. This 20 kb-fragment is present in the biotype 2-5 strains, but absent in most biotype 1A and B strains, and carries the toxin complex (TC) genes *tcaA*, *tcaB*, *tcaC *and *tccC *with homology to TC genes of *Photorhabdus luminescens*. However, the absence of TC-PAI_Ye _is not reflected by a loss of toxicity in case of subcutaneous infection, indicating the presence of yet unknown insecticidal determinants in *Y. enterocolitica *[15].

To investigate the genomic heterogeneity of the species *Y. enterocolitica*, we have chosen to sequence the genome of the low-pathogenicity strain W22703. We report the annotation of this second genome sequence of a *Y. enterocolitica *strain, and a detailed comparative genome analysis of the W22703 genome with that of strain 8081, a representative of the highly pathogenic biotype 1B group. The data obtained provide novel insights into the biology, metabolism, adaptation strategies and evolutionary relationships of *Y. enterocolitica*.

## Results

### General features

The shotgun sequencing of the *Y. enterocolitica *strain W22703 genome revealed a total number of 243656 reads with an average read length of 363. Assembly of 232502 reads resulted in 305 contigs larger and 705 contigs shorter than 1,000 base pairs (bp) with a median level of coverage in contigs > 5 kb of 16.49 (Additional file 1); one contig (1796) exceeds this coverage level more than twice (40x). The genome has an average G + C content of 46.9% (Table 1). Upon PEDANT based annotation [16] and search against a non-redundant protein database, 4003 genes corresponding to a coding density of 84.4% could be identified, but an unknown number of genes might have been missed due the short contigs not assembled (Additional file 2). The analysis also revealed at least 68 tRNA genes. The fewer number of tRNA genes compared to finished *Yersinia *genomes is probably due to collapsing of reads of the repeat sequences into fewer contigs [17]. The exact number of rRNA operons could not be estimated from this draft assembly, as reads from identical copies probably assemble into the same contigs. The risk of frameshifts due to sequencing errors in longer homo-oligomers was reduced by the high coverage of the assembly. We have determined 111 pairs of consecutive ORFs having best similarity to the same protein. However, this number also includes real pseudogenes not affected by any sequencing error.

**Table 1:** Genome features

Property	**W22703 pYV**^**-**^	***Y. enterocolitica *8081 **[18]
Size [bp]	<4,754,619	4,615,899
G + C content [%]	46.9	47.3
Number of contigs >1000 bp	305	2 (linear chromosome, plasmid pYV)
Number of CDs	>4,003	4,037
Coding density [%]	84.4	83.8
Average gene size	unknown	968
rRNA operons	unknown	7
tRNAs	≥ 68	81
Common IS elements (numbers)	IS911A, ISRM3, IS1328 (3), IS1329, IS1400, IS1666, IS1667 (4), IS1668, IS1669	
IS elements not shared	IS2A/D/F/H/I/K; iso-IS1N; IS1328; IS911B	IS3, IS4, IS1111, IS1222, IS1330, IS1541, IS1660 (6), IS1664, IS1665 (6), IS1669 (4), IS1664 (2), IS1222, IS3, IS Sod4
Prophage regions	2	4
Sequence coverage	16-fold	9-fold

### Genome comparison with strain 8081

**Figure 1:**
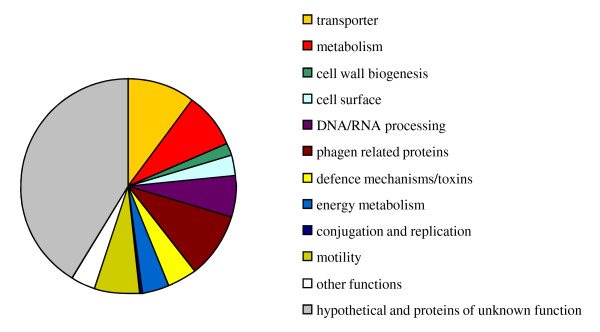
Functional categories of genes present in strain W22703 and absent in strain 8081

*Y. enterocolitica *8081 is one of three strains of this species whose sequences were available until February 2011 [18-20]. It belongs to the biotype 1B group with higher pathogenicity potential to humans than the biotype 2-5 group. To delineate the most relevant features of the W22703 genome, we decided to base our further analysis on a genome comparison between the shotgun sequence of strain W22703 and the linear genome sequence of *Y. enterocolitica *strain 8081. The alignment of both genomes using Mauve [21] shows long syntenic regions with few rearrangements and a general high sequence conservation, but also regions in both genomes that are not shared with the other (Additional file 3). Upon automatic and manual BLAST analysis, we revealed 550 genes present in the 8081 genome but absent in that of W22703, and 551 genes that are specific for W22703 with respect to strain 8081. The virulence plasmid pYV [22] was not considered here. Figure 1 shows the categories under which the W22703 genes absent in 8081 are summarized. Besides hypothetical genes and those of unknown function, the largest numbers of gene-encoded factors fall into the functional groups transporter, metabolism and DNA/RNA processing. The latter group comprises 18 regulatory genes. The motility and phage sections are mainly composed of the ancestral flagellar locus *flgII *(see below) and one specific prophage.

### Regions of difference

**Figure 2:**
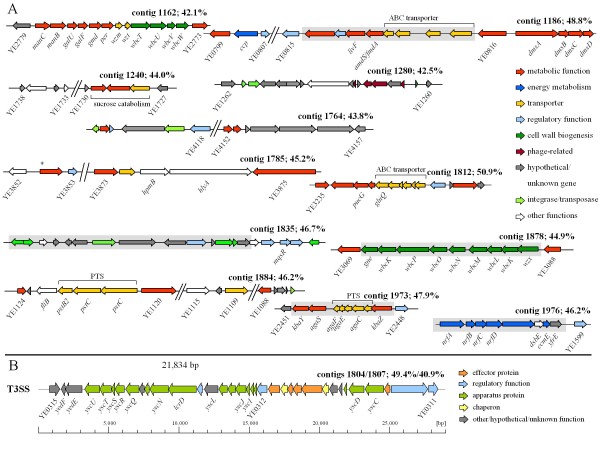
**Regions of strain W22703 without counterpart in strain 8081**. **A) contig 1162**: *manC*, mannose-1-phosphate guanylytransferase; *manB*, phosphomannomutase; *galU*/*galF*, UTP-glucose-1-phosphate uridylyltransferase; *gmd*, probable GDP-mannose 4,6-dehydratase; *per*, perosamine synthetase; *wbcT*/*U*/*V*/*W*, glycosyltransferase. **contig 1186**: *ccp*, cytochrome c peroxidase; *livF*, branched chain amino acid transporter; *amdS*/*fmdA*, acetamidase/formamidase; *dmsA/B/C*, anaerobic DMSO reductase; the gene right of YE0815 encodes a putative nitrilase/cyanide hydratase or apolipoprotein N-acyltransferase; gap ~7,100 bp. **contig 1240**: the gene right of YE1738 encodes a putative O-acetylhomoserine aminocarboxypropyltransferase; gap ~4,230 bp. **contig 1764**: the gene right of YE4152 encodes a GCN5-related N-acetyltransferase; gap ~39,960 bp. **contig 1785**: the gene right of YE3852 encodes a putative acyltransferase; *hpmB*, hemolysin activator; *hlyA*, hemolysin A; gap ~20,340 bp. **contig 1812**: *pucG*, purine catabolism protein (aminotransferase); *glnQ*, glutamine ABC transporter ATP-binding protein. **contig 1835**: *mqsR*, motility quorum-sensing regulator. **contig 1878**: *gne*, uridine diphosphatoacetylglucosamine epimerase; *wbcK*/*P*/*O*/*N*/*M*/*L*/*K*, glycosyltransferase; wzx, lipopolysaccharide O-unit flippase. **contig 1884**: gaps ~6,960/21,100 bp. **contig 1973**: *kbaY*, tagatose-1,6-bisphosphate aldolase; *agaS*, tagatose-6-phosphate ketose/aldose isomerase; *kbaZ*, tagatose 6-phosphate kinase. **contig 1976**: *nrfA-D*, formate-dependent nitrate reductase, cytochrome c; *dsbE*, hypothetical thiol:disulfide interchange protein; *ccmE*, cytochrome C biogenesis protein. Shaded regions: no counterparts were identified in *Y. pseudotuberculosis *or *Y. pestis *genomes. The GC-content of the regions is added to the contig number. Asterisk: low-temperature induced gene as identified by luciferase reportering [60]. **B) A novel T3SS (*ysa2*) of strain W22703 **YE0311 putatively encodes a two-component response regulator, the gene left of it a multi-sensor hybrid histidine kinase. Different GC contents were determined for the regions left and right of YE0312.

We then searched for regions of difference (ROD) between the genome sequences of 8081 and W22703. By definition, those ROD genes do not belong to the core genome of the two *Y. enterocolitica *strains compared here, but might constitute additional metabolic or virulence-associated properties contributing to the overall strain fitness. Twelve ROD present in strain W22703 are shown in Figure 2A. While the average GC-content of the W22703 genome sequence is 46.9%, the ROD on contigs 1240, 1162, 1764, 1812, and 1280 show an at least 2% higher or lower GC-content, suggesting their acquisition by lateral gene transfer (LGT) [23]. Phylogenetic tree analysis, however, revealed closely related genes of these contigs in other *Yersinia *strains, with the exception of contig 1280 that harbours phage related genes. The 8081 genes flanking the ROD might give additional information about the underlying recombination events. For example, a glycosyltransferase operon of 8081 (YE3070-YE3087) might have been replaced by a related operon on contig 1878 (or vice versa) that possibly contributes to O-antigen synthesis. A substitution of hypothetical genes by a non-homologous cluster of functionally unknown genes is observed in contig 1764. The ROD on contigs 1186, 1240, 1280 and 1973 obviously interrupt gene linearity with respect to the 8081 genome, indicating that loss and substitution, or insertion, of genes might have taken place in these cases. Contig 1280 harbours several phage-related genes and is therefore assumed to represent a second prophage region. Transposase genes were found in the 8081 genome between YE2773 and YE2779, a region covered by the LPS synthesis in W22703 (contig 1162), and a similar observation was made for the PTS encoding cluster on contig 1884. More functional details on these ROD are described below.

### Virulence genes or cluster present or absent in W22703 compared with 8081

While YGI-1, which is responsible for adhesion and includes a T4SS, is completely encoded on contig 1802, the high pathogenicity island (HPI) encoding yersiniabactin [24] is missing in the genome of strain W22703. In contrast, we identified 18 functions probably involved in defence or virulence mechanisms present in W22703, but absent in 8081 (Table 2). Two autotransporter or type V secretion proteins (contig 1177), one of them with homology to an AidA-like adhesin, might play a role in (non-mammalian) host-recognition by W22703. The insecticidal pathogenicity island TC-PAI_Ye _is characteristic for biotype 2-5 strains, but absent in biotype 1A and 1B strains with the exception of WA314 (biotype 1B, serotype O:8) [14,15]. Another homology group of TC genes was found to be prevalent among clinical biotype 1A strains [25]. Beside a second, non-clustered *tccC2 *locus [15], no further factors with homology to toxin complex genes could be identified in W22703.

**Table 2:** Predicted functions of genes present in W22703 and absent in 8081

Transporter	Contig
amino acids ABC transporters	1812, 1658^§^, 1393, 1802*, 1162*, 1660^§^, 1186, 1757
PTS	1973^§^, 1884
major facilitator superfamily	1950^§^, 1885
permeases	1802, 1240, 1885^§^, 1996
sodium/bile acid symporter family	1785^§^
efflux transporter, RND family	1328
divalent cation transporter	1165
iron complex transport system substrate-binding protein*	1360
magnesium transporter	1165

**Bioenergetic proteins**	**Contig**
cytochrome-c protein	1186, 1976^§^
nitrate reductase	1976^§^

**Conjugation/replication**	**Contig**
conjugation transfer protein, TraD family^§^	1170
MobA/MobL-like protein^§^	1170
type IV prepilin^§^	1170^§^
chromosome segregation ATPase	1804

**Metabolism**	**Contig**
amidase, hydantoinase/carbamoylase family protein	1812
serine-pyruvate transaminase	1812
serin protease	2008
transferase	1022
zinc-dependent protease	1432
tagatose-6-phosphate metabolism	1973
prolyl endopeptidase	1967^§^
oxidoreduktase	1963^§^
format hydrogenlyase	1947
acetamidase/formamidase	1186
multisensor hybrid histidine kinase	1807
iron-sulfur cluster repair di-iron protein	1794
GCN5-like N-acetyltransferases	1764, 1785^§^
L-carnitine-dehydratase*	1314
sucrose catabolism^§^	1240
glycogen phosphorylase	1240
FAD-dependent pyridine-nucleotide-disulphid-oxidoreductase	1218
anaerobic dimethyl-sulfoxide-reductase	1186
nitrilase/cyanid-hydratase/apolipoprotein N-acyltransferase	1186
thiol-disulfid exchange protein	1976^§^
carbonic-anhydrase^§^	1116
purine catabolism protein	1812
NAD-dependent aldehyde-dehydrogenase	1764^§^

**Cell wall biogenesis or surface proteins**	**Contig**
flagella gene cluster II*	1803, 1891
outer membrane protease	1116
small integrale membrane protein^§^	1246
perosamine synthetase	1162
surface antigen	1162
outer membrane protein	1802, 1177
pertactin-like protein	1146
TonB-dependent heme receptor	1757
phosphatidylinositol-diacylglycerol-lyase	1423
uridine-diphosphatoacetylglucosamine epimerase	1878
pili biogenesis and assembly	1847, 1393, 1882
cell wall-associated hydrolases	1386^§^, 1192
integral membrane protein CcmA	1766
lipopolysaccharide synthesis	1162, 1878
extracellular ligand-binding receptor	1186
lipoprotein	1803

**Defense mechanisms/toxins**	**Contig**
T3SS*	1804, 1807
bile salt/choloylglycin hydrolase (*bsh*)	1088
toxin-antitoxin system	1946, 1930
zonula occludens-toxin	1920
Rtx cluster *rtxH*, *rtxC*, *rtxA*	1867^§^
autotransporter cluster	1177
insecticidal toxins (TC-PAI_Ye_)	1579, 1975, 1854, 1855, 1856
small toxic polypeptide	1810
hemolysin; hemolysin activator protein	1785
colicin-E2/pyocin S2 immunity protein operon	1216
antibiotic acetyltransferase	1885^§^, 1921

**Proteins for DNA/RNA regulation and processing**	**Contig**
transcriptional regulator	1088*, 1894, 1885, 1825^§^, 1835^§^(2), 1764, 1557^§^, 1200^§^, 1171^§^, 1885(2) ^§^, 1891, 1803, 1802
RpiR-like transcriptional regulator	1812
CRP/FNR family transcriptional regulator*	1088
LuxR-like transcriptional regulator	1921
negative regulator of β-lactamase expression	1384
response regulator receiver protein	1367, 1186
methyltransferase	1835, 1170
DNA topoisomerase	1958, 1225
helix destabilizing protein	1920
superfamily I DNA helicase	1825
methionyl-tRNA-formyltransferase	1162
transcription repressor	1920
filamentation induced by cAMP protein Fic	1088
sensor protein with MASE1 domain	1432
NTP-binding protein	1116, 1170, 1897, 1882, 1763, 1760, 1837

**Others**	**Contig**
plasmid stabilisation system*	1835
resolvase	1404, 1835
secreted protein	1211, 1456
prophage region	1280, 1796,1920*

*absent in all *Yersinia *species sequenced until 12/2010

^§^absent in *Y. pestis *and *Y. pseudotuberculosis*, but present in other *Yersinia *species

To survive within its host organisms, *Y. enterocolitica *needs to overcome both cellular (hemocytic) and peptide-mediated components. A candidate of the former group is the exported repeats-in-toxin RtxA, a cytolytic toxin of approximately 3200 amino acids length encoded by contig 1867. It clusters with a putative Rtx activating protein, RtxC, and a peptide chain release factor 1, RtxH (Table 2). Among the yersiniae, only one *Y. enterocolitica *serotype O:3 strain and *Y. kristensenii *carry homologs of these proteins. The genome of W22703 encodes three two-partner secretion (TCS) systems involved in hemolysin release, one of which is absent in the 8081 genome. Three peptidases of W22703 might play a role in resistance towards antimicrobial peptides. W22703 also produces an antibacterial protein or bacteriocin that is absent in 8081. The pyocin locus on contig 1216 probably encodes killer proteins and a dual type immunity protein with domains similar to pyocin-E2 and colicin (S2). Its biological function, as well as that of a small toxic polypeptide (contig 1810), is yet unknown.

### Secretion/transfer systems and transporters

Two distinct, chromosomally located type three secretion systems (T3SS) mark one of the most striking differences between the two genomes. While *ysa *of 8081 is absent in W22703, this strain harbours another T3SS, which we termed *ysa2*, located on contigs 1804 and 1807 (Figure 2B). PCR targeting the flanking regions resulted in a ~200 bp fragment, justifying the link of both contigs. Sequence analysis of *ysa2 *revealed a mosaic structure with a G/C content of 49.4% between the flanking genes YE0315 and YE0312, and a G/C content of 40.9% between YE0312 and YE0311, indicating two independent LGT events. The whole cluster is collinear to respective regions in apathogenic yersiniae such as *Y. frederikseni *and *Y. intermedia*. We found homologs of the plasmid-encoded T3SS of *Y. pestis *and *Y. pseudotuberculosis*, and partial collinearity, with respect to the left part of this 29 kb island. The right part carries *yscC *and *yscD *homologs, but no homologs of YopB, YopD or LcrV involved in translocon formation [26]. The functionality of *ysa2 *is unknown. Of the two type 2 secretion system (T2SS) cluster *yst1 *and *yst2 *in 8081, only *yst2 *responsible for a general secretion pathway (GSP) is present in strain W22703. Contig 1170 encodes factors involved in conjugal DNA-transfer, namely TraD, a MobA/MobL protein, and a putative type IV prepilin that might contribute to LGT.

As iron is often a rate-limiting factor for pathogenic bacteria during infection, W22703 requires iron-scavenging systems for survival in the host. Beside the two iron and enterobactin transport systems within the PZ, those comprise a hemophore cluster for heme binding and uptake (Table 2), and a putative iron binding protein (contig 1360), the latter one absent in 8081.

We also identified eight ABC transporters in W22703, two phosphotransferase systems (PTS), four permeases, two major facilitators, a sodium:bile acid symporter, and other transporters listed in Table 2 or mentioned in the sections on metabolism and virulence. All of these are without counterparts in the genome of strain 8081. A glucitol/sorbitol-specific transporter (contig 1884; YE1093-YE1098) and a sorbose uptake system are also present in 8081, but not in *Y. pestis *and *Y. pseudotuberculosis*, and have homologs in all or most non-pathogenic species sequenced so far. A cellobiose uptake system was also identified (contig 1882). In total, our analysis identified a higher number of putative transporters with respect to strain 8081.

### Plasticity zone (PZ)

**Figure 3:**
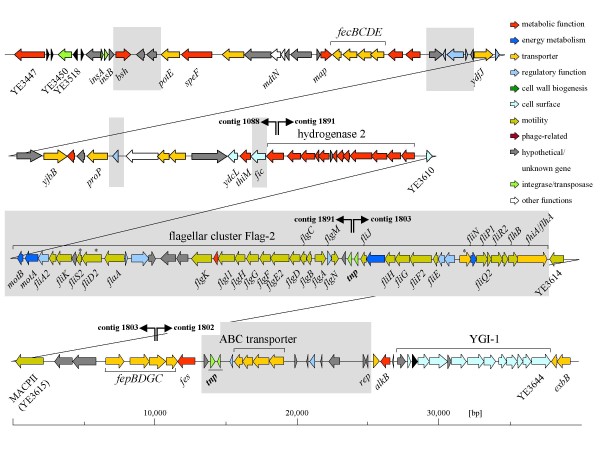
**Plasticity zone of strain W22703**. Genes and encoded proteins: *speF*, ornithine decarboxylase; *bsh*, chologlycin or bile salt hydrolase; *potE*, putrescine-ornithine antiporter; *mdtN*, multidrug resistance protein; *map*, methionine aminopeptidase; *ydfJ*, putative metabolite transporter; *yjbB*, putative Na^+^/Pi-cotransporter; *proP*, betain/proline transporter; *ydcL*, putative lipoprotein; *thiM*, hydroxyethylthiazole kinase; *fic*, filamentation induced by cAMP protein; *motA*/*motB*, flagellar motor proteins; *tnp*, transposase; MACPII, methyl-accepting chemotaxis protein; *fes*, enterochelin esterase; *virF*, virulence regulon transcriptional activator; *rep*, IncF plasmid RepFIB replicon; *alkB*, α-ketoglutarate-dependent dioxygenase; *exbB*, MotA/TolQ/ExbB proton channel family protein; asterisks: low-temperature induced genes as identified by luciferase reportering [60]. Shaded regions are absent in 8081. See text for more details.

The plasticity zone of strain 8081 ranges from YE3447 to YE3644 and has a total length of approximately 199 kb with 186 coding sequences (CDS). It was defined by Thomson *et al*. [18] as the largest region of species-specific genomic variation among *Y. enterocolitica *biotypes, and it is absent from *Y. pestis *and *Y. pseudotuberculosis*. Four contigs revealed to carry PZ genes. We linked contigs 1088/1891 and 1803/1802 due to the presence of truncated *hypF *and *fepG *genes, respectively, at their ends. The primer combination 5'-GTTTCTTTATGGGCGCG-3'/5'-TTGGCATGGAGGCCTG-3' hybridizing to the ends of contigs 1891 and 1803 resulted in a PCR product of approximately 1500 bp, thus allowing the linear reconstruction of the W22703-specific PZ (Figure 3). With a total length of ~ 142,000 kb, it is significantly shorter than that of 8081 and exhibits a comparably low density of virulence genes. Many discrete functional units of the 8081 PZ are indeed missing in the W22703 genome as confirmed by BLAST search of any PZ-encoded protein against the translated shotgun sequence. The most prominent ones are (i) the pathogenicity island YAPI_Ye _of 66 CDS including a putative hemolysin, a toxin/antitoxin system *ccdA*/*ccdB*, and a type IV pilus operon, (ii) the T3SS *ysa *important for pathogenicity in an mouse oral infection model [27] and (iii) the T2SS *yst1 *required for full virulence [28]. Further 8081 PZ genes absent in W22703 are the two putative two component-systems (TCS) YE3561/YE3563 and YE3578/YE3579, a chitinase (YE3576), a putative lipase (YE3614), a putative copper/silver efflux system (YE3626-YE3630), and the arsenic resistance operon. However, it is also worthy of note that PZ loci assumed or known to play a role in pathogenicity towards invertebrates or vertebrates are present in the W22703 genome. Examples are the YGI-1 mentioned above, the hydrogenase 2- (*hyb*-) locus, *fecBCDE *encoding an iron transporter, the ferric enterbactin transport system *fepBDGC*/*fes*, and *proP *encoding a betain/proline transporter involved in osmoprotection and osmoregulation.

The recently identified flagellar cluster Flg-2 [29] within the PZ of W22703 is absent in the genome of 8081. It comprises 44 genes encoding factors for the flagellar motility apparatus and for flagellar biosynthesis, but lacks chemotaxis genes (Figure 3). A region of approximately 11,300 bp flanked by a transposase gene and the replicon of an IncF plasmid RepFIB comprises an ABC transporter and a regulatory gene; however, the functionality of this region, which is unique with respect to all *Yersinia *sequences available so far, is in doubt due to its low coding density.

### Surface components

The main flagella and chemotaxis gene cluster I (*flg-1*) of 8081 is present in W22703 (contigs 1361, 1428, 1469 and 1890), but only one of two type-1 fimbrial operons was found (contig 1271; YE0782-YE0786). The surface-exposed lipopolysaccharide (LPS) molecule constituting the serotype O:9 O-antigen is synthesized by the O-polysaccharide gene cluster (contig 1162) [30]; a second glycosyltransferase gene cluster is located on contig 1878 (Figure 2A). The role of the O-polysaccharide and the outer core hexasaccharide in resistance of *Y. enterocolitica *to human complement and polymyxin B has been described recently [30].

### Metabolism

Several enzymatic activities common to both *Y. enterocolitica *genomes compared here are involved in nitrogen metabolism. Examples are the capability to catalyse urease that is encoded by seven genes on contig 1225. The assimilation of the urease product ammonia for amino acid and nucleotide synthesis is then achieved by glutamine synthase. Two ornithine decarboxylases forming putrescine, and a putrescine/ornithine antiporter are encoded on the PZ and also contribute to amino acid metabolism.

Like 8081, strain W22703 carries the *cel *gene cluster for cellulose production (contig 1798) and the genes *mdoC, mdoG *and *mdoH *for osmoregulated periplasmic glucan (OPG) biosynthesis (contig 1967). Both the capability to produce cellulose and to synthesize OPGs have been lost or inactivated in *Y. pestis *and *Y. pseudotuberculosis*. OPG mutants exhibit deficiencies in virulence, biofilm formation and antibiotic resistance, as well as hypersensitivity towards bile salts [18].

The capability to utilize propanediol in a cobalamin (vitamin B_12_)-dependent manner is encoded on contigs 2012, 1667, 1476, 1555, 1999, and 1235, and the respective *cob/cbi/pdu *genes are collinear to the 8081 genes YE2707-YE2750. In line with the yersiniae core genome, *ttr *genes responsible for tetrathionate reduction are present (contig 1975), and the *eut *genes allowing B_12_-dependent ethanolamine utilization are absent. The *mtn *genes located on contig 1812 are involved in methionine salvage. This cobalamin-dependent pathway recycles methylthioadenosine derived from sperimidine, spermin and *N*-acylhomoserine lactone synthesis. The hydrogenases Hyd-4 encoded within the *hyf *locus (contigs 1162, 1947) and Hyd-2 within the *hyb *locus (PZ; contig 1891, Figure 3) are also present in W22703.

#### Distinct metabolic properties of W22703

W22703 is endowed with several metabolic enzymes that are unique in comparison to strain 8081 (Figure 2A), among them a serine-pyruvate transaminase involved in glycine-, serine- and threonine-metabolism (contig 1812). The reductases encoded on contigs 1186 and 1976 suggest that W22703, but not 8081, is able to use nitrate and dimethylsulfoxide (DMSO) as alternative electron acceptor under anaerobic conditions. In contrast, pathways for trimethylamine and thiosulfate oxidation are present in both genomes. Beside the DMSO reductase, contig 1186 harbours another ROD encoding an ABC transporter, a putative nitrilase or cyanide hydratase that catalyzes nitrile into amino acids and ammonium or hydrogen cyanid into formamide, and a putative acetamidase or formamidase. Contig 1973 carries a gene cluster enabling W22703 to uptake *N*-acetylgalactosamine that is then isomerized to tagatose. In addition to YE0550A-YE0555, we identified a second operon for sucrose utilization on contig 1240.

#### Metabolic pathways lost in W22703

We identified few enzymes or capabilities that are missing in W22703 in comparison to 8081 (Table 3). Examples are the absence of a chitinase, and of the lipase YE3614 that is probably responsible for the lipase negative reaction of W22703 as a biotype 2 strain [31]. In addition to the arsenic resistance operon on YAPI_Ye_, homologs of a second operon with this function (YE3364-YE3366) are absent in W22703.

**Table 3:** Predicted functions of genes present in 8081 and absent in W22703

Transporter	Gene
copper/silver efflux system	YE3626-YE3630
multidrug efflux protein	YE0443
ABC transporter	YE0801-YE0804; YE0818-YE0821
sugar permease	YE2605-YE2609
phosphohistidinoprotein-hexose phosphotransferase component of PTS system (Hpr)	YE1207
glucoside specific PTS system	YE2103-2107
membrane transport protein	YE2029, YE3695
molybdate ABC transporter permease protein	YE2912
cation efflux system	YE3628

**Bioenergetic proteins**	**Gene**
sulfite reductase subunit protein	YE1597
NADH:flavin oxidoreductase/NADH oxidase family protein	YE3034

**Conjugation/replication**	**Gene**
conjugal transfer protein	YE1177-YE1178
type 4 prepilin-like proteins leader peptide processing enzyme	YE3574
integrating conjugative element protein	YE3484, YE3495
TraG-family protein	YE3493

**Metabolism**	**Gene**
chitinase	YE3576
lipase	YE3614
zinc metalloprotease	YE4052
hydrolase	YE0306
dehydrogenase/reductase	YE0341, YE1369-YE1370
aldo/keto reductase	YE3031
oxidoreductase	YE0484
D-serine dehydatase	YE0800
glycerol kinase	YE0824
UDP-sugar hydrolase/5'-nucleotidase periplasmic precursor	YE3066
acetyltransferase	YE1087, YE2245
fucose isomerase *fucI*	YE0825

**Cell wall biogenesis or surface proteins**	**Gene**
adhesin/invasin	YE0694
YGI-2 (putative glycolipoprotein)	YE0894-YE0912
type-1 fimbrial operon	YE1111-YE1114
type-4 pilus	YE3498-YE3506
*ysaE *(invasin)	YE3549
*yadA*	YE1873
lipopolysaccharide synthesis	YE3070-3087
lipoprotein	YE0982, YE1234-YE1236, YE1871, YE3483, YE4156
N-acetylglucosamine-binding protein	YE3576 (*chiY*), YE2830, YE3411, YE3494A
acyl-UDP-N-acetylglucosamine O-acyltransferase, *lpxA2*	YE2286

**Defense mechanism/toxins**	**Gene**
high pathogenicity island HPI (Yersiniabactin)	YE2611-YE2622
HlyA-like hemolysin and transporter (TPS)	YE2407, YE2408
hemolysin	YE3454
hemophore	YE0125-YE0126
heme acquisition system	YE0123-YE0126
T3SS effector protein	YE2447
autotransporter	YE1372, YE2049
*ccdA*/*ccdB*, toxin/antitoxin system	YE3480-YE3481; YE2032-2033
bifunctional antitoxin/transcriptional repressor RelB	YE0510, YE0647
arsenic resistance operon	YE3364-YE3366; YE3472-YE3478
metal resistance protein	YE1299
*ysa*, T3SS	YE3533-YE3561
*yst1*, T2SS	YE3564-YE3575
iron-sulfur cluster assembly protein	YE1059
metallo-β-lactamase superfamily protein	YE2635
cytonecrosis factor-like toxin	YE2091
actin-ADP-ribosylating toxin	YE0115
antibiotic biosynthesis monooxygenase	YE0340
Rho-activating domain of cytotoxic necrotizing factor	YE2091

**Proteins for DNA/RNA regulation and processing**	**Gene**
TCS	YE3561/YE3563; YE3578/YE3579
LuxR family transcription regulatory protein	YE0039, YE0343, YE1026, YE1165, YE2050-YE2051, YE3033
transcriptional regulatory protein	YE0826, YE4110
DNA-binding protein	YE1102, YE1172, YE1825, YE3496A, YE4129
Cro/CI family transcriptional regulator	YE1175A
plasmid-related transcriptional repressor protein	YE1182
GntR-family regulatory protein	YE2670
DNA helicase	YE3514
DEAD-like helicase	YE2632

**Others**	**Gene**
YGI-3, putative integrated plasmid	YE0975-YE0993
YGI-4, putative integrated plasmid	YE1170-YE1183
YAPI_Ye_	YE3450-YE3515
prophage region YE98	YE0854-YE0888
prophage region YE185	YE1667-YE1693
prophage region YE200	YE1799-YE1819
prophage region YE250	YE2292-YE2363
zinc-binding protein	YE0683
ATP/GTP-binding protein	YE0986, YE0990, YE3513
plasmid stabilisation protein	YE0510A, YE1110
chromosome partitioning system	YE3515

### Dynamic genomes: further regions absent in W22703 or 8081

Whole genome comparison allows to follow the dynamic processes by which genomes separate from a common ancestor. In addition to genomic islands or clusters already mentioned above, the genomes of the two *Y. enterocolitica *strains compared here differ by a set of regions, indicating the dynamic of sequence acquisition and loss. The prophage regions YE98 (YE0854-YE0888), YE185 (YE1667-YE1693), YE200 (YE1799-YE1819) and YE250 (YE2292-YE2363) of strain 8081 are absent in strain W22703 that, however, carries another prophage of 37 CDS in contig 1796. Then, the *Yersinia *genome islands YGI-2, YGI-3 and YGI-4 [18] are missing in W22703. YGI-2 carries genes for the synthesis, modification, and export of an outer membrane anchored glycolipoprotein. Of this island, only homologs of YE0912 encoding a 2,5 diketo-D-gluconic acid reductase B and of YE0911 encoding a 3-oxo-acyl-(acyl carrier protein) synthase II are present in W22703. On contig 1854, we identified two homologs of YE0979, which encodes a DNA-binding protein, and the hypothetical gene YE0980 from YGI3 harbouring a putative integrated plasmid.

## Discussion

The genome analysis and genome comparison performed here intended to contribute to a better understanding of the ecology, pathogenicity and evolution of *Y. enterocolitica*. Adding, rearranging and reducing or losing DNA has been proposed as the general recipe for *Yersinia *genome evolution when eight less-pathogenic strains had been compared [17].

Several ROD shown in Figure 2A-B and Figure 3 might be recent acquisitions due to the significant deviation of their G/C content, while others might have been acquired early after separation of both strains, or indicate regions that have been lost or substituted in 8081. Beside the virulence plasmid, the PZ is a second example for the acquisition of virulence genes. Remarkably, this region is approximately 55 kb shorter in W22703 and lacks several determinants proposed or known to contribute to pathgenicity towards humans. Together with the presence of a large ancient flagellar gene cluster and a region of obvious genetic degeneration, this finding strongly reflects the lower virulence potential of W22703 in comparison to 8081 [32], and confirms the importance of this region for the manifestation of virulence properties of *Y. enterocolitica *[18].

Other (virulence) regions absent in W22703 might have been acquired by 8081 after separation of both strains. The novel T3SS (*ysa2*) that is absent in the pathogenic species *Y. pestis *and *Y. pseudotuberculosis*, but present in apathogenic species such as *Y. intermedia*, *Y. frederiksenii *and *Y. kristensenii*, might play a role in the interaction with non-mammalian hosts. The TC proteins have been shown to be secreted upon activity of the plasmid-encoded T3SS of *Y. pestis *[33]. Since we used a pYV-free W22703 derivative to demonstrate TC-based insecticidal activity of this strain [13], *ysa2 *is a candidate for TcaA secretion by W22703. This finding supports the assumption that T3SS are not unique vehicles for delivering anti-vertebrate factors but ancient secretion systems for the transport of effector molecules across host membranes, with the potential to play a role in a wide range of bacteria-host interactions [34]. Together with a number of putative virulence factors of W22703 (Table 2), *ysa2 *contributes to the pathosphere of yersiniae, a concept hypothesizing that all of the pathogenic genes shared by enteric bacteria form a "pool" [35].

The pathogen *Y. enterocolitica *has a complex life cycle encompassing aquatic and biological environments. Due to its known capability to interact with invertebrates and mammals, it exhibits a multiphasic phenotype upon colonizing and potentially killing more than one host species [36]. Although little is known about putative signals and regulatory circuits required to switch or modulate necessary changes from one state to the other, candidates are genes listed in Table 2 or induced at low temperature [37].

Although some of their functions remain to be experimentally confirmed, the metabolic pathways present in strain W22703 confirm the relevance of several metabolic traits for gut-adapted *Y. enterocolitica*. Examples are cobalamin-dependent utilization of propanediol, hydrogenase activities, cellulose production, tetrathionate reduction and ornithine decarboxylase activity, all of which are absent, lost or inactivated in systemic *Y. pestis *and *Y. pseudotuberculosis *[17,18]. Interestingly, tetrathionate that acts as a terminal electron acceptor during anaerobic degradation of 1,2-propanediol or ethanolamine is formed in the inflamed gut upon infection [38]. The hydrogenases Hyd-4 and Hyd-2 might contribute to the adaptation of yersiniae to gut environments [39,40]. Two additional reductases of W22703 allowing to use nitrate and DMSO are in line with this assumption. A potentially insect-specific resistance mechanism and/or catabolic trait of strain W22703 is provided by the ROD of contig 1186 (Figure 2A) inserted between YE0815 and YE0816. It encodes an acetamidase/formamidase, a branched chain amino acid transporter, an ABC transporter and a putative nitrilase/cyanide hydratase. These predicted functions point to a role of this chromosomal region in the acquisition of nitrogen sources. Indeed, insects such as *Zygaena filipendulae *produce cyanogenic glucosides that might then be used by W22703 as nitrogen source via a metabolic route of bacteria that includes cyanide nitrilase, hydratase and formate dehydrogenase activities [41-43]. Although speculative so far, the functions of contig 1186 might represent a further determinant contributing to invertebrate host adaptation of strain W22703. The operon on contig 1973 including a PTS is responsible for tagatose utilization; this metabolic trait is not only common to human intestinal bacteria, but was found to be specifically induced during insect infection by *P. luminescens *[44,45]. Interestingly, the PZ gene *bsh *encodes a chologlycin hydrolase or bile salt hydrolase (Figure 3) that catalyzes the deconjugation of conjugated bile salts to liberate amino acids and free primary bile acids [46].

Recently, a genome comparison between the genomes of the insect pathogen *P. luminescens *and *Y. enterocolitica *8081 revealed a huge number of common genes that might contribute to the adaptation of yersiniae to invertebrate hosts [37]. Interestingly, we identified nearly all of these factors also in the genome of W22703, underlining the assumption that *Y. enterocolitica *strains share the capability to interact with nematodes or insects.

## Conclusion

Although further genome sequences are required to learn more about the evolution of *Y. enterocolitica *strains, this study indicates that beside the *Yersinia *virulence plasmids, the highly flexible PZ indeed contributes to the acquisition of determinants that might increase the pathogenicity towards humans. On the other hand, insecticidal toxins, the novel T3SS or specific metabolic properties might play a crucial role for the adaptation of *Y. enterocolitica *strains to non-mammalian hosts.

## Methods

### Bacterial strains and growth conditions

*Y. enterocolitica *W22703(pYVe227) is a nalidixic acid-resistant (Nal^R^) restriction mutant (Res-Mod') isolated from strain W227 [47]. A plasmidless isogenic derivative (W22703 pYV^-^) was used. To avoid contaminations and to validate the strain cultured for DNA isolation, strain W22703 pYV^- ^was streaked from a glycerol stock on *Yersinia *selective agar plates (CIN agar base; Becton Dickison, Heidelberg, Germany). A single colony was used for inoculation of Luria-Bertani (LB) broth (10 g l^-1 ^tryptone, 5 g l^-1 ^yeast extract, and 5 g l^-1 ^NaCl) containing 20 μg ml^-1 ^nalidixic acid, and the culture was grown for twelve hours at a selective temperature of 15°C. When the culture had reached stationary phase, aliquots were plated in parallel on LB and *Yersinia *seletive agar plates. PCRs targeting W22703-specific genes *tcaA*, *tcaC *and two genes of Flg-2 were performed as a further control.

### General molecular techniques

DNA and RNA manipulation was performed according to standard procedures [48]. To isolate chromosomal DNA, 1.5 ml of a bacterial culture was centrifuged, and the sediment was resuspended in 400 μl of lysis buffer (100 mM Tris pH 8.0, 5 mM EDTA, 200 mM NaCl). After incubation for 15 min on ice, 10 μl of 10% SDS and 5 μl of proteinase K (10 mg/ml) were added, and the sample was incubated overnight at 55°C. The chromosomal DNA was then precipitated with 500 μl of isopropanol, washed in ethanol, dried, and dissolved in 500 μl of TE buffer (10 mM Tris-HCl, 1 mM Na_2_EDTA, pH 7.4) containing 1 μl of RNase (10 mg/ml). Polymerase chain reactions (PCR) were carried out with Taq polymerase (Fermentas, Vilnius, Lithunia) and the following programme: one cycle at 95°C for 2 min; 30 cycles at 95°C for 10 sec, at the appropriate annealing temperature for 30 sec, at 72°C for 45 sec to 180 sec depending on the expected fragment length; one cycle at 72°C for 10 min. 4 μl of chromosomal DNA (100 ng ml^-1^) was used as template for PCR amplification, and the GeneRuler DNA mix (Fermentas) served as DNA ladder. For gap closure, the following oligonucleotides were used (targeted contigs): 5'-CAACATTAAATCACGAAGG-3'/5'-TTAGTACAAATACCGATGG-3' (1804/1807); 5'-GTTTCTTTATGGGCGCG-3'/5'-TTGGCATGGAGGCCTG-3' (1891/1803); 5'-TAACCTCTAGCGCGG-3'/5'-CCCCGATAGTTCTGG-3' (1088/1891).

### Genome sequencing and accession

High throughput sequencing of a shotgun library was done on the GS FLX system (Roche, 454 Life Sciences, Branford, USA) using the Titanium series with approximately 20-fold coverage, and assembly were performed by Eurofins MWG GmbH, Ebersberg, Germany. According to the newly defined standards for classification of genome sequences [49], the *Y. enterocolitica *genome sequence belongs to the category "Annotation-Directed Improvement". The EMBL accession numbers for the sequences reported in this paper are FR718488-FR718797. The raw sequence data files are deposited in the ENA trace archive as ERP000495. The annotated sequence is available under the URL address http://pedant.gsf.de.

### Genome annotation and analyses

The PEDANT software system (http://pedant.gsf.de; [50]) was used for automatic genome sequence analysis and annotation [16]. Protein coding genes were predicted using the GeneMarkS software program using default settings [51]. Biochemical pathway prediction and reconstruction were performed using the KEGG [52], BRENDA [53], and Microbes online [54] databases. tRNAs were identified using tRNAscanSE [55], rRNA homologs with blastn [56]. Additional manual homology searches of predicted proteins were performed by BLAST analysis (http://www.ncbi.nlm.nih.gov/BLAST/) to ascribe a protein function or domain.

Comparison with the genome sequence of *Y. enterocolitica *8081 (EMBL accession numbers are AM286415 for the chromosome and AM286416 for the virulence plasmid pYV; PEDANT database name is p3_p190_Yer_enter) was performed using the *Y. enterocolitica *Blast Server from the Sanger Institute (http://www.sanger.ac.uk/cgi-bin/blast/submitblast/yersinia). The criterion applied was an 80% identity of the amino acid sequence. Incomplete proteins encoded on contig ends were considered to be present in strain W22703 if the lacking sequence could be identified on another contig. Genome sequences of *Yersinia *strains were obtained from the NCBI database and compared using the homepage http://www.microbesonline.org/. Protein sequence alignment was done with the ClustalW program [57]. Phylogenetic trees for ROD and T3SS have been automatically calculated using the the software PhyloGenie [58] and the default parameters according to its documentation. We used NCBI nr [59] as reference database and excluded proteins of unclassified taxa.

## Abbreviations

ABC transporter: ATP-binding cassette transporter; DMSO: dimethylsulfoxide; LGT: lateral gene transfer; PTS: phosphotransferase system; ROD: region(s) of difference; TCS: two-partner secretion system; T3SS: type three secretion system; T2SS: type two secretion system; TCS: two component system; YGI: *Yersinia *genome island.

## Authors' contributions

TMF supervised the study, and drafted the manuscript. KB analysed the annotated genome, MS closed contig gaps, and TR was responsible for automatic genome sequence analysis and annotation. All authors read and approved the final manuscript.

## Supplementary Material

Additional file 1 FR accession numbers of all W22703 contigs Click here for file

Additional file 2 W22703 gene names, locus tags, and protein accession numbers. Click here for file

Additional file 3 **Mauve-type genome alignment between the reference genome of strain 8081 (chromosome and plasmid; top) and draft genome of strain W22703 (contigs; bottom)**. Red lines indicate chromosome and contig borders. Similar regions are indicated by frames and assigned to each other by connecting lines. The degree of sequence similarity is shown within each region as similarity plot. Click here for file

## References

[B1] Sulakvelidze A Yersiniae other than *Y. enterocolitica*, *Y. pseudotuberculosis*, and *Y. pestis*: the ignored species Microbes Infect 2000 2 497 513 10.1016/S1286-4579(00)00311-7 10865195

[B2] Sprague LD Neubauer H *Yersinia aleksiciae *sp. nov Int J Syst Evol Microbiol 2005 55 831 835 10.1099/ijs.0.63220-0 15774670

[B3] Sprague LD Scholz HC Amann S Busse HJ Neubauer H *Yersinia similis *sp. nov Int J Syst Evol Microbiol 2008 58 952 958 10.1099/ijs.0.65417-0 18398201

[B4] Merhej V Adekambi T Pagnier I Raoult D Drancourt M *Yersinia massiliensis *sp. nov., isolated from fresh water Int J Syst Evol Microbiol 2008 58 779 784 10.1099/ijs.0.65219-0 18398169

[B5] Hurst MR Becher SA Young SD Nelson TL Glare TR *Yersinia entomophaga *sp. nov. isolated from the New Zealand grass grub *Costelytra zealandica* Int J Syst Evol Microbiol 2010 20495033 10.1099/ijs.0.024406-0

[B6] Murros-Kontiainen AE Fredriksson-Ahomaa M Korkeala H Johansson P Rahkila R Bjorkroth J *Yersinia nurmii *sp. nov Int J Syst Evol Microbiol 2010 10.1099/ijs.0.024836-0 21037032

[B7] Murros-Kontiainen AE Johansson P Niskanen T Fredriksson-Ahomaa M Korkeala H Bjorkroth J *Yersinia pekkanenii *sp. nov Int J Syst Evol Microbiol 2010 10.1099/ijs.0.019984-0 21037033

[B8] Bottone EJ *Yersinia enterocolitica*: overview and epidemiologic correlates Microbes Infect 1999 1 323 333 10.1016/S1286-4579(99)80028-8 10602666

[B9] Wauters G Kandolo K Janssens M Revised biogrouping scheme of *Yersinia enterocolitica* Contrib Microbiol Immunol 1987 9 14 21 3665492

[B10] Tennant SM Grant TH Robins-Browne RM Pathogenicity of *Yersinia enterocolitica *biotype 1A FEMS Immunol Med Microbiol 2003 38 127 137 10.1016/S0928-8244(03)00180-9 13129647

[B11] Schubert S Rakin A Karch H Carniel E Heesemann J Prevalence of the "high-pathogenicity island" of *Yersinia *species among *Escherichia coli *strains that are pathogenic to humans Infect Immun 1998 66 480 485 9453599 10.1128/iai.66.2.480-485.1998 PMC107931

[B12] Wren BW The yersiniae - a model genus to study the rapid evolution of bacterial pathogens Nat Rev Microbiol 2003 1 55 64 10.1038/nrmicro730 15040180

[B13] Bresolin G Morgan JA Ilgen D Scherer S Fuchs TM Low temperature-induced insecticidal activity of *Yersinia enterocolitica* Mol Microbiol 2006 59 503 512 10.1111/j.1365-2958.2005.04916.x 16390445

[B14] Spanier B Starke M Higel F Scherer S Fuchs TM *Yersinia enterocolitica *infection and *tcaA*-dependent killing of *Caenorhabditis elegans* Appl Environ Microbiol 2010 76 6277 6285 10.1128/AEM.01274-10 20639372 PMC2937509

[B15] Fuchs TM Bresolin G Marcinowski L Schachtner J Scherer S Insecticidal genes of *Yersinia *spp.: taxonomical distribution, contribution to toxicity towards *Manduca sexta *and *Galleria mellonella*, and evolution BMC Microbiol 2008 8 214 10.1186/1471-2180-8-214 19063735 PMC2613401

[B16] Frishman D Albermann K Hani J Heumann K Metanomski A Zollner A Mewes HW Functional and structural genomics using PEDANT Bioinformatics 2001 17 44 57 10.1093/bioinformatics/17.1.44 11222261

[B17] Chen PE Cook C Stewart AC Nagarajan N Sommer DD Pop M Thomason B Thomason MP Lentz S Nolan N Genomic characterization of the *Yersinia *genus Genome Biol 2010 11 R1 10.1186/gb-2010-11-1-r1 20047673 PMC2847712

[B18] Thomson NR Howard S Wren BW Holden MT Crossman L Challis GL Churcher C Mungall K Brooks K Chillingworth T The complete genome sequence and comparative genome analysis of the high pathogenicity *Yersinia enterocolitica *strain 8081 PLoS Genet 2006 2 e206 10.1371/journal.pgen.0020206 17173484 PMC1698947

[B19] Wang X Li Y Jing H Ren Y Zhou Z Wang S Kan B Xu J Wang L Complete genome sequence of a *Yersinia enterocolitica *"Old World" (3/O:9) strain and comparison with the "New World" (1B/O:8) strain J Clin Microbiol 2011 10.1128/JCM.01921-10 PMC3122819 21325549

[B20] Batzilla J Hoper D Antonenka U Heesemann J Rakin A Complete genome sequence of *Yersinia enterocolitica *subsp. palearctica serogroup O:3 J Bacteriol 2011 21296963 10.1128/JB.01484-10 PMC3133053

[B21] Darling AC Mau B Blattner FR Perna NT Mauve: multiple alignment of conserved genomic sequence with rearrangements Genome Res 2004 14 1394 1403 10.1101/gr.2289704 15231754 PMC442156

[B22] Mulder B Michiels T Simonet M Sory MP Cornelis G Identification of additional virulence determinants on the pYV plasmid of *Yersinia enterocolitica *W227 Infect Immun 1989 57 2534 2541 2545628 10.1128/iai.57.8.2534-2541.1989 PMC313482

[B23] Hacker J Blum-Öhler G Mühldorfer I Tschape H Pathogenicity islands of virulent bacteria: structure, function and impact on microbial evolution Mol Microbiol 1997 23 1089 1097 10.1046/j.1365-2958.1997.3101672.x 9106201

[B24] Carniel E Guilvout I Prentice M Characterization of a large chromosomal "high-pathogenicity island" in biotype 1B *Yersinia enterocolitica* J Bacteriol 1996 178 6743 6751 8955291 10.1128/jb.178.23.6743-6751.1996 PMC178570

[B25] Tennant SM Skinner NA Joe A Robins-Browne RM Homologues of insecticidal toxin complex genes in *Yersinia enterocolitica *biotype 1A and their contribution to virulence Infect Immun 2005 73 6860 6867 10.1128/IAI.73.10.6860-6867.2005 16177365 PMC1230942

[B26] Cornelis GR The *Yersinia *Ysc-Yop 'type III' weaponry Nat Rev Mol Cell Biol 2002 3 742 752 10.1038/nrm932 12360191

[B27] Haller JC Carlson S Pederson KJ Pierson DE A chromosomally encoded type III secretion pathway in *Yersinia enterocolitica *is important in virulence Mol Microbiol 2000 36 1436 1446 10.1046/j.1365-2958.2000.01964.x 10931293

[B28] Iwobi A Heesemann J Garcia E Igwe E Noelting C Rakin A Novel virulence-associated type II secretion system unique to high-pathogenicity *Yersinia enterocolitica* Infect Immun 2003 71 1872 1879 10.1128/IAI.71.4.1872-1879.2003 12654803 PMC152056

[B29] Bresolin G Trcek J Scherer S Fuchs TM Presence of a functional flagellar cluster Flag-2 and low-temperature expression of flagellar genes in *Yersinia enterocolitica *W22703 Microbiology 2008 154 196 206 10.1099/mic.0.2007/008458-0 18174138

[B30] Skurnik M Biedzka-Sarek M Lubeck PS Blom T Bengoechea JA Perez-Gutierrez C Ahrens P Hoorfar J Characterization and biological role of the O-polysaccharide gene cluster of *Yersinia enterocolitica *serotype O:9 J Bacteriol 2007 189 7244 7253 10.1128/JB.00605-07 17693522 PMC2168460

[B31] Robins-Browne RM Doyle MP, Beuchat LR, Montville TJ Yersinia enterocolitica Food microbiology - Fundamentals and frontiers 1997 Washington D.C., ASM Press 192 215

[B32] Sory MP Cornelis G *Yersinia enterocolitica *O:9 as a potential live oral carrier for protective antigens Microb Pathog 1988 4 431 442 10.1016/0882-4010(88)90028-9 3143043

[B33] Gendlina I Held KG Bartra SS Gallis BM Doneanu CE Goodlett DR Plano GV Collins CM Identification and type III-dependent secretion of the *Yersinia pestis *insecticidal-like proteins Mol Microbiol 2007 64 1214 1227 10.1111/j.1365-2958.2007.05729.x 17542916

[B34] ffrench-Constant R Waterfield N Daborn P Joyce S Bennett H Au C Dowling A Boundy S Reynolds S Clarke D *Photorhabdus*: towards a functional genomic analysis of a symbiont and pathogen FEMS Microbiol Rev 2003 26 433 456 10.1111/j.1574-6976.2003.tb00625.x 12586390

[B35] Burland V Shao Y Perna NT Plunkett G Sofia HJ Blattner FR The complete DNA sequence and analysis of the large virulence plasmid of *Escherichia coli *O157:H7 Nucleic Acids Res 1998 26 4196 4204 10.1093/nar/26.18.4196 9722640 PMC147824

[B36] Thomson NR Clayton DJ Windhorst D Vernikos G Davidson S Churcher C Quail MA Stevens M Jones MA Watson M Comparative genome analysis of *Salmonella *Enteritidis PT4 and *Salmonella *Gallinarum 287/91 provides insights into evolutionary and host adaptation pathways Genome Res 2008 18 1624 1637 10.1101/gr.077404.108 18583645 PMC2556274

[B37] Heermann R Fuchs TM Comparative analysis of the *Photorhabdus luminescens *and the *Yersinia enterocolitica *genomes: uncovering candidate genes involved in insect pathogenicity BMC Genomics 2008 9 40 10.1186/1471-2164-9-40 18221513 PMC2266911

[B38] Winter SE Thiennimitr P Winter MG Butler BP Huseby DL Crawford RW Russell JM Bevins CL Adams LG Tsolis RM Gut inflammation provides a respiratory electron acceptor for *Salmonella* Nature 2010 467 426 429 10.1038/nature09415 20864996 PMC2946174

[B39] Maier RJ Olczak A Maier S Soni S Gunn J Respiratory hydrogen use by *Salmonella enterica *serovar Typhimurium is essential for virulence Infect Immun 2004 72 6294 6299 10.1128/IAI.72.11.6294-6299.2004 15501756 PMC523013

[B40] Olson JW Maier RJ Molecular hydrogen as an energy source for *Helicobacter pylori* Science 2002 298 1788 1790 10.1126/science.1077123 12459589

[B41] Kunz DA Wang CS Chen JL Alternative routes of enzymic cyanide metabolism in *Pseudomonas fluorescens *NCIMB 11764 Microbiology 1994 140 1705 1712 10.1099/13500872-140-7-1705 8075806

[B42] Zagrobelny M Bak S Ekstrom CT Olsen CE Moller BL The cyanogenic glucoside composition of *Zygaena filipendulae *(Lepidoptera: Zygaenidae) as effected by feeding on wild-type and transgenic lotus populations with variable cyanogenic glucoside profiles Insect Biochem Mol Biol 2007 37 10 18 10.1016/j.ibmb.2006.09.008 17175442

[B43] Zagrobelny M Bak S Olsen CE Moller BL Intimate roles for cyanogenic glucosides in the life cycle of *Zygaena filipendulae *(Lepidoptera, Zygaenidae) Insect Biochem Mol Biol 2007 37 1189 1197 10.1016/j.ibmb.2007.07.008 17916505

[B44] Bertelsen H Andersen H Tvede M Fermentation of D-tagatose by human intestinal bacteria and diary lactic acid bacteria Microbial Ecology in Health and Disease 2001 13 87 95 10.1080/089106001300136147

[B45] Münch A Stingl L Jung K Heermann R *Photorhabdus luminescens *genes induced upon insect infection BMC Genomics 2008 9 229 18489737 10.1186/1471-2164-9-229 PMC2422844

[B46] Jones BV Begley M Hill C Gahan CG Marchesi JR Functional and comparative metagenomic analysis of bile salt hydrolase activity in the human gut microbiome Proc Natl Acad Sci USA 2008 105 13580 13585 10.1073/pnas.0804437105 18757757 PMC2533232

[B47] Cornelis G Colson C Restriction of DNA in *Yersinia enterocolitica *detected by recipient ability for a derepressed R factor from *Escherichia coli* J Gen Microbiol 1975 87 285 291 1095683 10.1099/00221287-87-2-285

[B48] Sambrook J Russell DW Molecular cloning: a laboratory manual 2001 3 Cold Spring Harbor Laboratory, Cold Spring Harbor, N. Y

[B49] Chain PS Grafham DV Fulton RS Fitzgerald MG Hostetler J Muzny D Ali J Birren B Bruce DC Buhay C Genomics. Genome project standards in a new era of sequencing Science 2009 326 236 237 10.1126/science.1180614 19815760 PMC3854948

[B50] Walter MC Rattei T Arnold R Guldener U Munsterkotter M Nenova K Kastenmuller G Tischler P Wolling A Volz A PEDANT covers all complete RefSeq genomes Nucleic Acids Res 2009 37 D408 411 10.1093/nar/gkn749 18940859 PMC2686588

[B51] Besemer J Lomsadze A Borodovsky M GeneMarkS: a self-training method for prediction of gene starts in microbial genomes. Implications for finding sequence motifs in regulatory regions Nucleic Acids Res 2001 29 2607 2618 10.1093/nar/29.12.2607 11410670 PMC55746

[B52] Kanehisa M Araki M Goto S Hattori M Hirakawa M Itoh M Katayama T Kawashima S Okuda S Tokimatsu T Yamanishi Y KEGG for linking genomes to life and the environment Nucleic Acids Res 2008 36 D480 484 10.1093/nar/gkm882 18077471 PMC2238879

[B53] Barthelmes J Ebeling C Chang A Schomburg I Schomburg D BRENDA, AMENDA and FRENDA: the enzyme information system in 2007 Nucleic Acids Res 2007 35 D511 514 10.1093/nar/gkl972 17202167 PMC1899097

[B54] Dehal PS Joachimiak MP Price MN Bates JT Baumohl JK Chivian D Friedland GD Huang KH Keller K Novichkov PS MicrobesOnline: an integrated portal for comparative and functional genomics Nucleic Acids Res 38 D396 400 10.1093/nar/gkp919 19906701 PMC2808868

[B55] Lowe TM Eddy SR tRNAscan-SE: a program for improved detection of transfer RNA genes in genomic sequence Nucleic Acids Res 1997 25 955 964 10.1093/nar/25.5.955 9023104 PMC146525

[B56] Altschul SF Madden TL Schaffer AA Zhang J Zhang Z Miller W Lipman DJ Gapped BLAST and PSI-BLAST: a new generation of protein database search programs Nucleic Acids Res 1997 25 3389 3402 10.1093/nar/25.17.3389 9254694 PMC146917

[B57] Thompson JD Higgins DG Gibson TJ CLUSTAL W: improving the sensitivity of progressive multiple sequence alignment through sequence weighting, position-specific gap penalties and weight matrix choice Nucleic Acids Res 1994 22 4673 4680 10.1093/nar/22.22.4673 7984417 PMC308517

[B58] Frickey T Lupas AN PhyloGenie: automated phylome generation and analysis Nucleic Acids Res 2004 32 5231 5238 10.1093/nar/gkh867 15459293 PMC521674

[B59] Sayers EW Barrett T Benson DA Bolton E Bryant SH Canese K Chetvernin V Church DM DiCuccio M Federhen S Database resources of the National Center for Biotechnology Information Nucleic Acids Res 2011 39 D38 51 10.1093/nar/gkq1172 21097890 PMC3013733

[B60] Bresolin G Neuhaus K Scherer S Fuchs TM Transcriptional analysis of long-term adaptation of *Yersinia enterocolitica *to low-temperature growth J Bacteriol 2006 188 2945 2958 10.1128/JB.188.8.2945-2958.2006 16585756 PMC1447024

