# Supplementary figures and images for: Shotgun sequencing of Yersinia enterocolitica strain W22703 (biotype 2, serotype O:9): genomic evidence for oscillation between invertebrates and mammals
Source: BMC Genomics. 2011 Mar 31;12:168. doi: 10.1186/1471-2164-12-168 (PMC3079665; doi:10.1186/1471-2164-12-168)

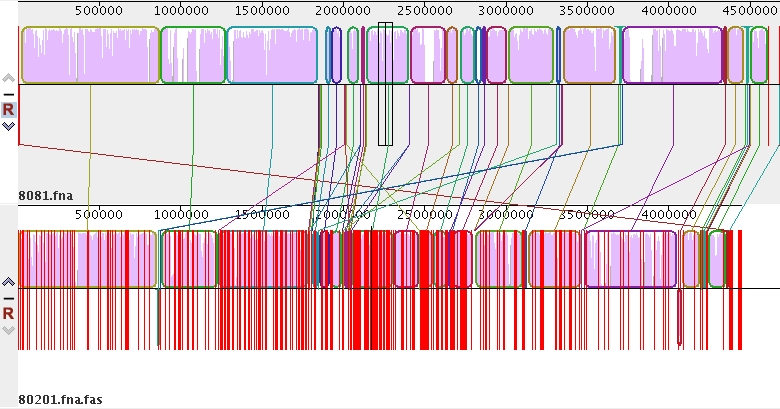

Supplement: Additional file 3 — Mauve-type genome alignment between the reference genome of strain 8081 (chromosome and plasmid; top) and draft genome of strain W22703 (contigs; bottom). Red lines indicate chromosome and contig borders. Similar regions are indicated by frames and assigned to each other by connecting lines. The degree of sequence similarity is shown within each region as similarity plot. [file 1471-2164-12-168-S3.BMP]
